# Impact of administering umbilical cord‐derived mesenchymal stem cells to cynomolgus monkeys with endometriosis

**DOI:** 10.1002/rmb2.12540

**Published:** 2023-09-08

**Authors:** Shunichiro Tsuji, Takeo Mukai, Hideaki Tsuchiya, Chizuru Iwatani, Akiko Nakamura, Tokiko Nagamura‐Inoue, Takashi Murakami

**Affiliations:** ^1^ Department of Obstetrics and Gynecology Shiga University of Medical Science Otsu Japan; ^2^ Department of Pediatrics The University of Tokyo Hospital Bunkyo‐ku, Tokyo Japan; ^3^ Research Center for Animal Life Science Shiga University of Medical Science Otsu Japan; ^4^ Department of Cell Processing and Transfusion, The Institute of Medical Science The University of Tokyo Minato‐ku, Tokyo Japan

**Keywords:** CA‐125 antigen, endometriosis, intraperitoneal injections, *Macaca fascicularis*, mesenchymal stem cells

## Abstract

**Purpose:**

This study aimed to explore whether umbilical cord‐derived mesenchymal stem cells (UC‐MSCs) could be used as a therapeutic resource for endometriosis.

**Methods:**

Of seven cynomolgus monkeys with endometriosis, five were administered UC‐MSCs (intervention group) and two were administered saline (control group). First, intravenous US‐MSC treatment was administered for three months. Second, weekly intravenous US‐MSC administration combined with monthly intraperitoneal US‐MSC administration was conducted for 3 months. Finally, weekly intraperitoneal US‐MSC administration was conducted for 3 months. The dose of UC‐MSCs was set to 2 × 10^6^ cells/kg for all administration routes. Laparoscopic findings and serum cancer antigen 125 (CA125) levels were also evaluated. The Revised American Society for Reproductive Medicine classification was used for laparoscopic evaluation.

**Results:**

Laparoscopic findings showed exacerbation of endometriosis after intraperitoneal UC‐MSC administration, although no changes were observed in the control group. Intravenous UC‐MSC administration decreased the level of CA125 in all monkeys; however, the difference was not significant. Intraperitoneal UC‐MSC administration significantly exacerbated endometriosis compared with intravenous administration (*p* = 0.02).

**Conclusions:**

This study revealed that intraperitoneal UC‐MSC administration exacerbates endometriosis in a nonhuman primate model of the disease.

## 1 INTRODUCTION

Endometriosis is a chronic pelvic inflammatory disease caused by ectopic endometrium.^1^ Inflammation can lead to dysmenorrhea, chronic pelvic pain, and infertility. Hormonal therapy is a known method for relieving these symptoms in women with endometriosis.^2^ However, it is less useful in patients who wish to conceive because hormonal therapy suppresses ovulation. Therefore, establishing novel treatments for endometriosis that do not suppress ovulation is desirable.

Many recent reports have indicated that mesenchymal stem cells (MSCs), also called mesenchymal stromal cells, can be applied for gynecological disorders.^3^, ^4^ However, their efficacy in treating endometriosis remains unclear. Umbilical cord (UC)‐derived MSCs (UC‐MSCs) inhibit endometriotic cell proliferation in vitro.^5^, ^6^ In rodent models, adipose tissue‐derived stem cells inhibited the growth of endometriosis‐like lesions.^7^, ^8^ In contrast, Abomaray et al. demonstrated that MSCs derived from ectopic and eutopic endometrial tissues are pluripotent and support the growth of endometriotic lesions through immunosuppressive effects.^9^ The same authors also concluded that the use of adipose tissue‐derived MSCs should not be considered a potential therapy for endometriosis.^10^ Furthermore, Li et al. demonstrated that MSCs derived from the endometrium promote fibrogenesis in ovarian endometriomas.^11^

To resolve these arguments, experiments with MSCs administered to monkeys, a nonhuman primate model, with spontaneous endometriosis are needed. The safety of administering human MSCs to nonhuman primates has been confirmed.^12^ Therefore, in this study, we administered UC‐MSCs to cynomolgus monkeys (*Macaca fascicularis*) with spontaneous endometriosis and examined the effects.

## 2 MATERIALS AND METHODS

### 2.1 Ethical approval and animals

This study was conducted at the Research Center for Animal Life Science at Shiga University of Medical Science with approval from the Animal Care and Use Committee of Shiga University of Medical Science (approval number: 2021‐3‐8). This study was carried out in strict accordance with the Fundamental Guidelines for Proper Conduct of Animal Experiments and Related Activities in Academic Research Institutions, regulated by the Ministry of Education, Culture, Sports, Science, and Technology of Japan.

Monkeys (*Macaca fascicularis*) with spontaneous endometriosis were housed individually in cages. Drinking water was provided ad libitum. The breeding environment has been described in our previous report.^13^ All monkeys were confirmed free of herpes B virus, hepatitis E virus, *Mycobacterium tuberculosis*, *Shigella* spp., *Salmonella* spp., and *Entamoeba histolytica*.

### 2.2 Laparoscopy

Laparoscopic observation of the monkeys was performed before intervention and after each treatment, as described in our previous report.^13^ Laparoscopy was performed under general anesthesia using ketamine and xylazine. A laparoscopic port was placed at the midline of the lower abdomen, and another port for the operating rod was placed in the lower right abdomen. A laparoscope (LA‐6500; Machida Endoscope Co. Ltd., Chiba, Japan) was connected to a recording device (SHIMIZU Laboratory Supplies Co., Ltd., Kyoto, Japan). The lesion size was measured using operating rod‐tip markings (Figure S1).

### 2.3 Establishment of UC‐MSCs

This study was approved by the Ethics Committees of the Institute of Medical Science, University of Tokyo (approval number: 31‐2). UCs were collected after informed consent was obtained from pregnant women planning to undergo a cesarean section. The fresh and frozen–thawed UC tissues were minced into 1–2‐mm^3^ fragments.^14^ Tissue fragments were cultured in α‐minimal essential medium (Wako Pure Chemical Industries, Ltd., Osaka, Japan) supplemented with 10% fetal bovine serum and antibiotics–antimycotics (Antibiotic–Antimycotic 100×; Life Technologies, Carlsbad, CA, USA) at 37°C under 5% CO_2_ using a Cellamigo® stainless steel mesh (Tsubakimoto Chain Co., Osaka, Japan) for improved explant isolation. Fibroblast‐like adherent cells that migrated from the tissue fragments were harvested using TrypLE Select (Life Technologies) and defined as passage‐1 cells. The harvested cells were passaged until passage 4, after which the cells were used for UC‐MSC experimental analyses. UC‐MSCs were cryopreserved in a cryoprotectant.

### 2.4 Flow cytometry analysis

Standard flow cytometry was performed to determine the presence of MSC markers, as defined by the International Society for Cellular Therapy.^15^ UC‐MSCs were stained using a Human MSC Analysis kit (BD Biosciences, Franklin Lakes, NJ, USA) containing the following mouse monoclonal antibodies, which are positive markers for MSCs: fluorescein isothiocyanate (FITC)‐conjugated anti‐human CD90; phycoerythrin (PE)‐conjugated anti‐human CD105; allophycocyanin (APC)‐conjugated anti‐human CD73; FITC‐conjugated anti‐human CD44 (BD Biosciences), and PE‐conjugated anti‐HLA‐ABC (BD Biosciences). Additionally, UC‐MSCs were stained with FITC‐conjugated anti‐HLA‐DR (BD Biosciences), FITC‐conjugated anti‐human CD34 (BD Biosciences), PE‐conjugated anti‐human CD11b (BD Biosciences), PE‐conjugated anti‐human CD19 (BD Biosciences), or APC‐conjugated anti‐CD45 (BD Biosciences); these are negative markers for MSCs. Propidium iodide was used to identify and exclude dead cells using flow cytometry, as previously described.^16^

### 2.5 Administration of UC‐MSCs

**FIGURE 1:**
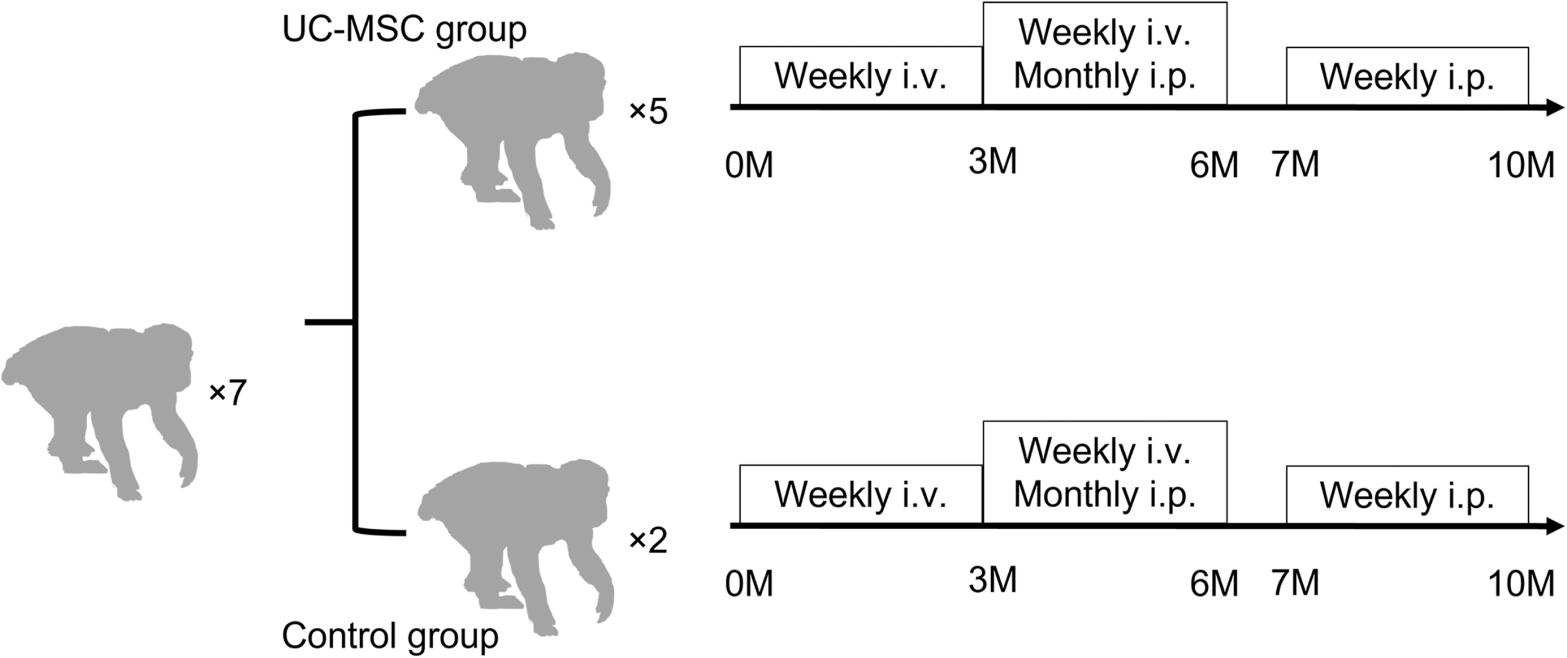
Simple schematic representation of the protocol used in this study. The umbilical cord‐derived mesenchymal stem cell (UC‐MSC) dose was 2 × 10^6^ cells/kg for both intravenous and intraperitoneal administration. M, months.

Five of the seven monkeys were assigned to the intervention group that received UC‐MSCs. The remaining two monkeys were used as controls; to whom saline was administered instead of UC‐MSCs. Allocation to intervention and control groups was determined based on laparoscopic findings to determine whether the monkeys had endometriosis before study initiation. Monkeys with relatively severe laparoscopic findings were assigned to the intervention group, and one monkey with mild laparoscopic findings was also assigned to the intervention group, as we considered that it would be important to evaluate whether UC‐MSCs could treat severe endometriosis when considering clinical applications in humans. UC‐MSCs were dissolved in physiological saline and administered at a dosage determined with reference to a prior report that confirmed the efficacy and safety of administering human MSCs to monkeys.^12^ The dose was 2 × 10^6^ cells/kg body weight for intravenous and intraperitoneal administration. For the first 3 months, UC‐MSCs were administered intravenously once per week. However, since intravenous administration only was ineffective, weekly intravenous injections were administered in conjunction with intraperitoneal injections once per month for the next 3 months. After a 1‐month interval, weekly intraperitoneal injections were administered for the last 3 months to assess the direct effect (Figure 1).

### 2.6 Evaluation of endometriosis

Laparoscopy is commonly used to assess endometriosis, using the Revised American Society for Reproductive Medicine (r‐ASRM) Classification of Endometriosis.^17^ In previous reports, the R‐ASRM score has also been used to determine endometriosis staging in monkeys.^18^, ^19^

Cancer antigen 125 (CA125) is a well‐known serum marker of endometriosis.^20^ The abovementioned laparoscopic findings and CA125 concentrations are reportedly useful for diagnosing endometriosis in monkeys.^19^ Blood was collected during laparoscopy under general anesthesia but not during the menstrual phase. The samples were sent to SRL, Inc. (Tokyo, Japan) to measure serum CA125 concentration.

### 2.7 Statistical analysis

Statistical analyses were performed using GraphPad Prism ver. 7 (GraphPad Software, Inc., San Diego, CA, USA). The D'Agostino–Pearson test was used to evaluate data distribution. Mean values were used in the analysis of normally distributed data. Median values were used in the analysis of non‐normally distributed data. For multiple comparisons, one‐way analysis of variance was used, followed by Dunn's post‐hoc test to determine statistical significance. Statistical significance was set at *p* < 0.05.

## 3 RESULTS

### 3.1 Establishment of UC‐MSCs

**FIGURE 2:**
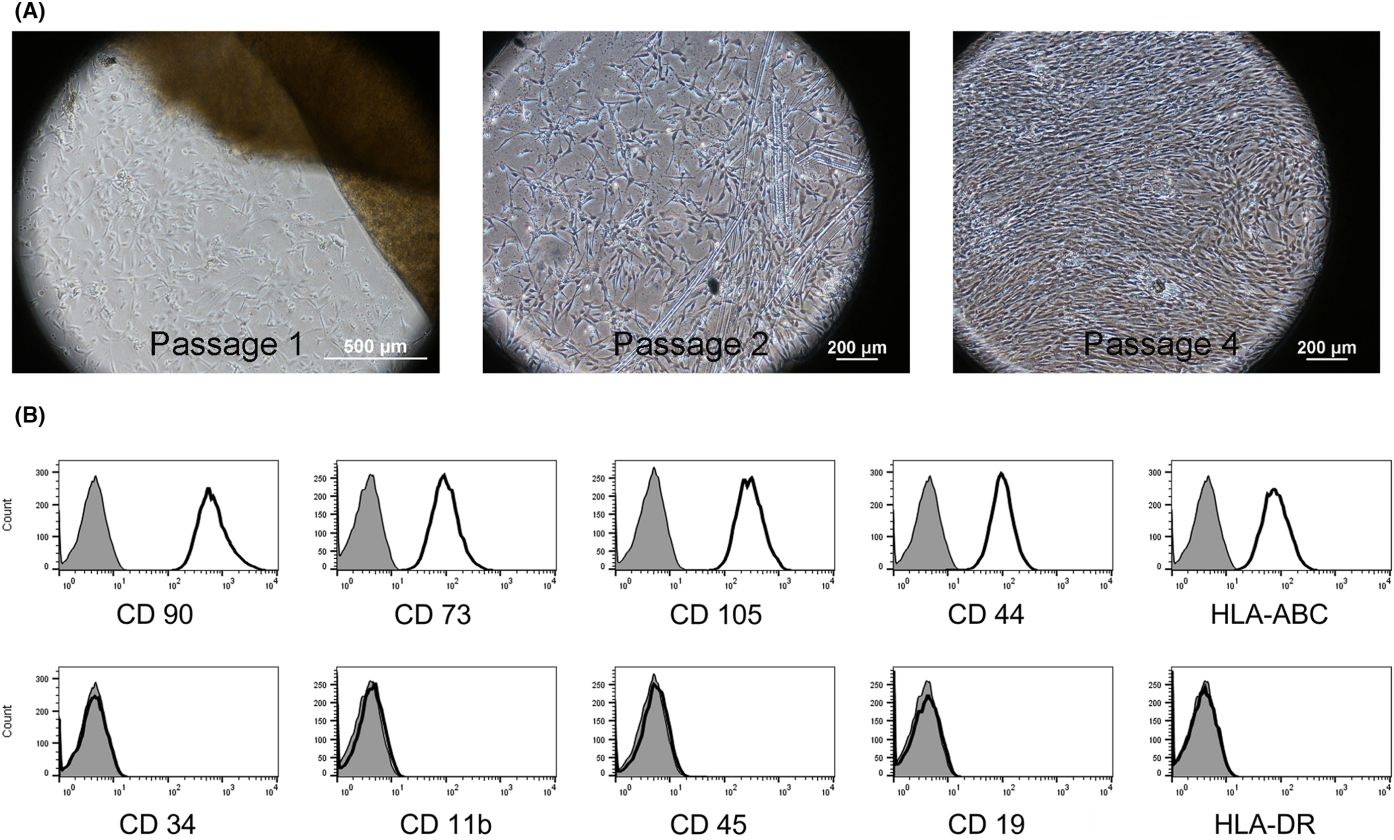
Characterization of umbilical cord‐derived mesenchymal stem cells (UC‐MSCs). (A) UC‐MSCs with spindle shapes isolated using the explant method. (B) Flow cytometry analysis shows that UC‐MSCs are positive for CD90, CD73, CD105, CD44, and HLA‐ABC and negative for CD 34, CD11b, CD45, CD19, and HLA‐DR.

All UC‐MSCs were spindle‐shaped, plastic‐adherent cells (Figure 2A). Analysis of the surface markers of UC‐MSCs showed that the cells were all positive for CD73, CD105, CD90, HLA‐ABC, and CD44 and negative for CD45, CD34, CD11b, CD 19, and HLA‐DR, as defined by the International Society for Cell Therapy (Figure 2B). Furthermore, these markers did not change, even after passage 4.

### 3.2 Laparoscopic findings

All cynomolgus monkeys with endometriosis exhibited regular menstrual cycles. Four monkeys were from Vietnam, one was from China, one from Indonesia, and one from the Research Center for Animal Life Science at the Shiga University of Medical Science (Table 1).

**TABLE 1:** Background of cynomolgus monkeys with endometriosis.

	Identification number	Origin	Age (years)	Body weight (kg)	Herpes B virus
Intervention group	I‐1	RCALS	17.9	4.1	Negative
	I‐2	Indonesia	17.2	2.4	Negative
	I‐3	Vietnam	17.1	2.9	Negative
	I‐4	Vietnam	16.2	3.4	Negative
	I‐5	Vietnam	11.1	3.6	Negative
Control group	C‐1	China	20.9	4.1	Negative
	C‐2	Vietnam	14.2	3.2	Negative

Abbreviation: RCALS, Research Center for Animal Life Science at Shiga University of Medical Science.

**FIGURE 3:**
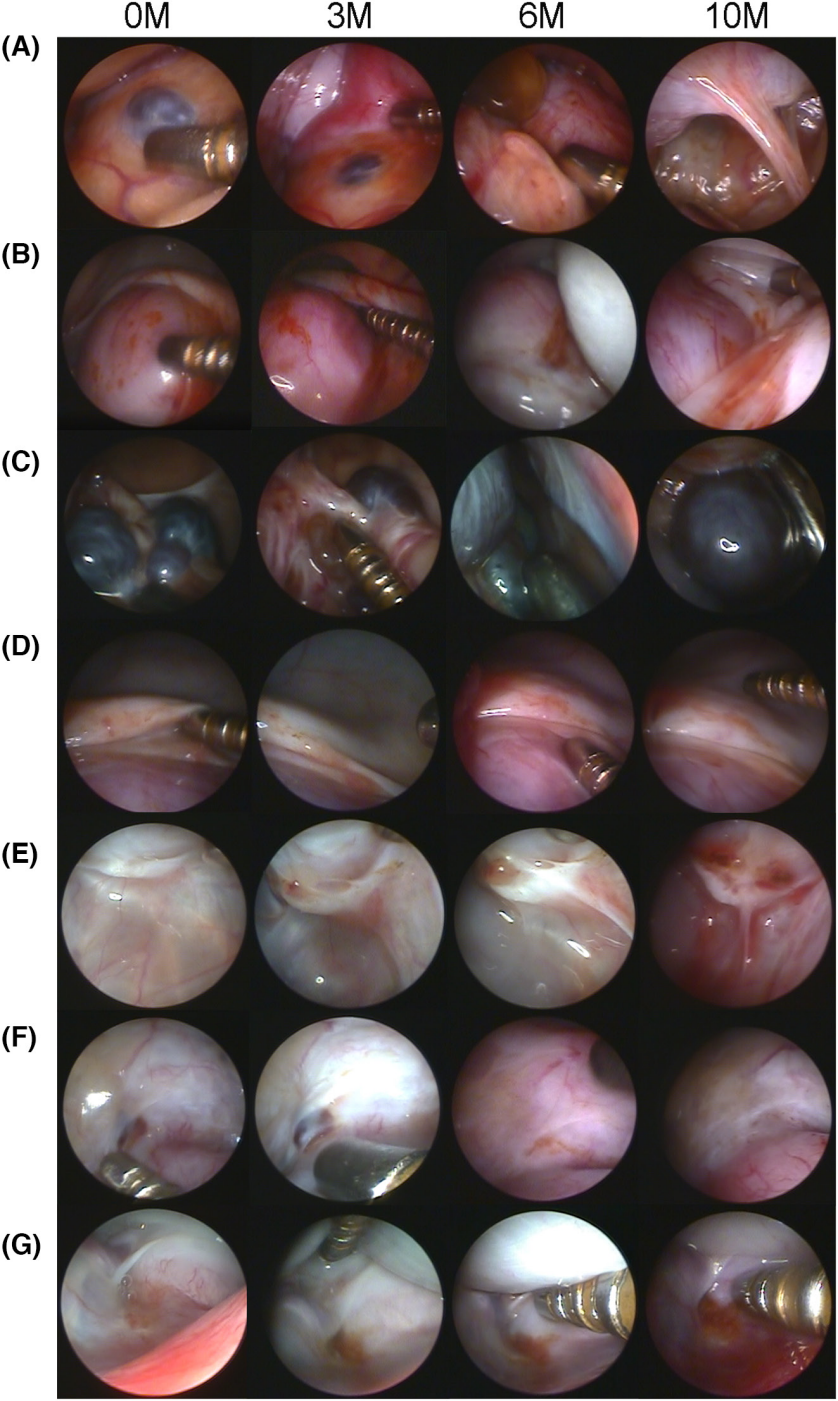
Laparoscopic findings during this study. (A) Adhesion of the uterus and omentum with a black lesion was observed in monkey I‐1. However, it was difficult to observe the lesion due to the severity of the adhesion after 6 months. (B) The uterus of monkey I‐2 demonstrated a red lesion; however, it was difficult to observe the uterus due to adhesion after 6 months. (C) The right adnexa of monkey I‐3 showed a black lesion. The lesion shrank after 3 months; however, it expanded after 6 months. (D) The red lesion of monkey I‐4 in the vesicouterine pouch expanded mildly during the experiment. (E) A red lesion and peritoneal thickening were observed in the pouch of Douglas in monkey I‐5. The lesion gradually expanded, and the peritoneum thickened markedly after 6 months. (F,G) Red lesions were observed in the vesicouterine pouches of monkeys C‐1 and C‐2. The size of the lesions remained unchanged throughout the experiment.

The laparoscopic findings were as follows: In monkey I‐1, adhesions of the uterus and omentum with black lesions were observed before the intervention. The black lesion on the omentum had not changed in size after 3 months. In contrast, adhesions around the uterine fundus progressed after 6 months and were so severe that they could not be observed laparoscopically after 10 months. In monkey I‐2, red lesions were observed on the serosal surface of the uterus prior to intervention. At 6 months, adhesions around the uterus appeared, and at 10 months, it was necessary to move the adhesion sites with an operating rod to observe the fundus of the uterus (Figure 3B). In monkey I‐3, three cystic black lesions were observed around the right adnexa. At 3 months, two of these had nearly disappeared, and one had shrunk. However, the black lesions had regrown at 6 months and increased in size at 10 months (Figure 3C). In monkey I‐4, red lesions were observed in the vesicouterine pouch, which expanded slightly during the course of the experiment (Figure 3D). In monkey I‐5, small red lesions and mild peritoneal thickening were initially observed in the pouch of Douglas. Over time, the lesions gradually expanded, and the peritoneum thickened, particularly after intraperitoneal UC‐MSC administration (Figure 3E). In contrast, in monkeys C‐1 and C‐2 (control groups), red lesions were initially observed on the pelvic peritoneal surface; however, no significant changes were observed in these lesions throughout the study period (Figure 3F,G).

### 3.3 R‐ASRM score

**FIGURE 4:**
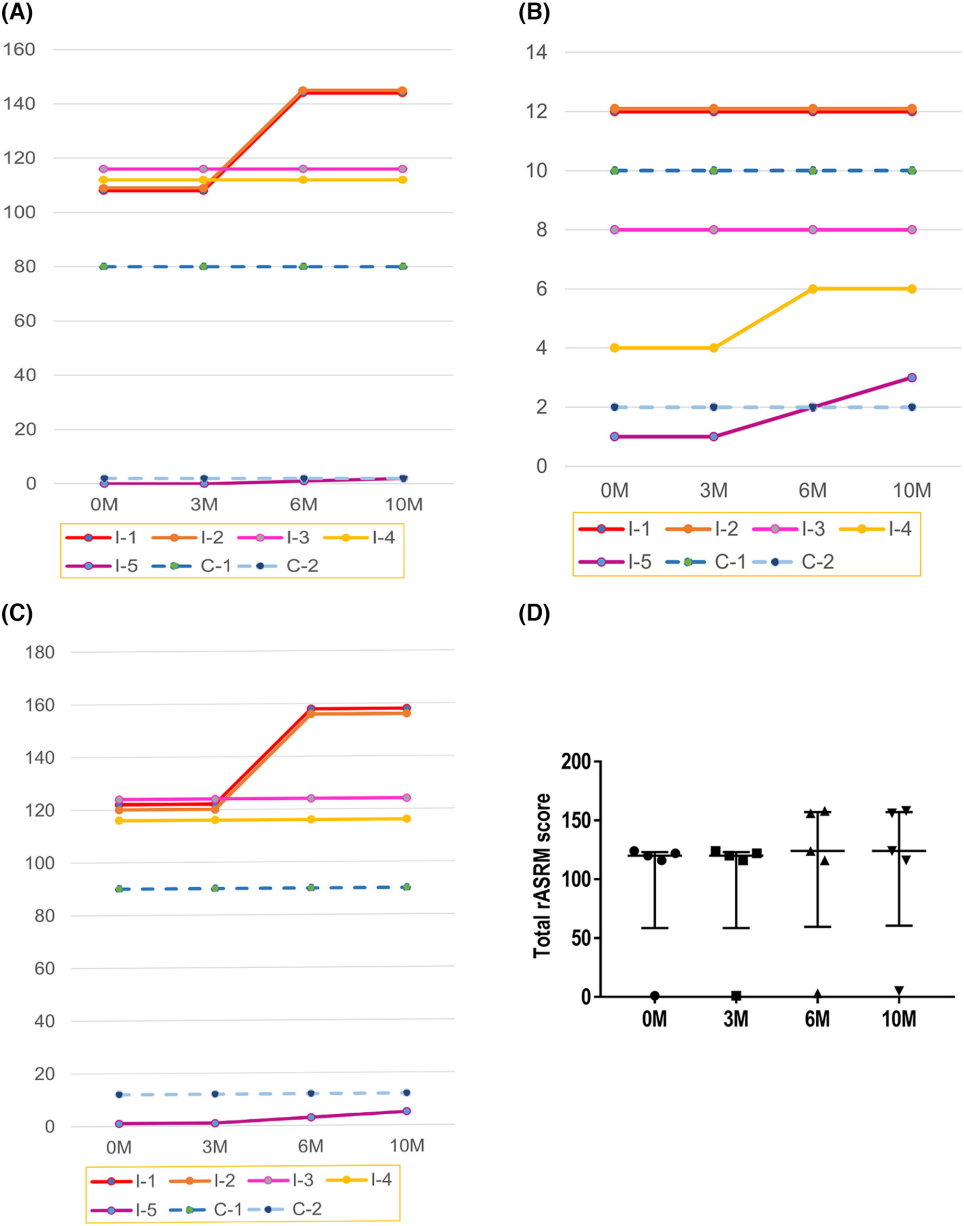
Changes according to the Revised American Society for Reproductive Medicine (r‐ASRM) classification for endometriosis. The solid line indicates the intervention group, and the dashed line represents the control group. (A) Adhesion score. (B) Size score. (C) Total r‐ASRM score. (D) Comparison of the total r‐ASRM score during the study.

In the r‐ASRM classification, the adhesion scores of I‐1 and I‐2 were 108 both before intervention and at 3 months; however, they increased to 144 in both monkeys by 6 and 10 months. Monkeys I‐3 and I‐4 showed scores of 116 and 112, respectively, which did not change throughout the study. The adhesion score for monkey I‐5 was zero before the experiment and at 3 months, one at 6 months, and two at 10 months. In the control group, the adhesion scores for monkeys C‐1 and C‐2 before the intervention were 80 and 2, respectively, and did not change throughout the experiment (Figure 4A).

By r‐ASRM classification, the size scores of monkeys I‐1, I‐2, and I‐3 before the experiment began were 12, 12, and 8, respectively. The scores did not change throughout the study period. The size score of monkey I‐4 was four before the experiment and at 3 months; however, it increased to six at 6 months and remained six at 10 months. The score of monkey I‐5 also increased throughout the study: one before the experiment, one at 3 months, two at 6 months, and three at 10 months. In contrast, in the control group, the size scores of monkeys C‐1 and C‐2 did not change throughout the study (Figure 4B).

The total score in the intervention group increased, except for those of monkeys I‐3 and I‐4. The scores of monkeys C‐1 and C‐2 in the control group did not change (Figure 4C). The median total scores also did not differ significantly between the intervention and control groups (Figure 4D).

### 3.4 Body weight and CA125 levels

**FIGURE 5:**
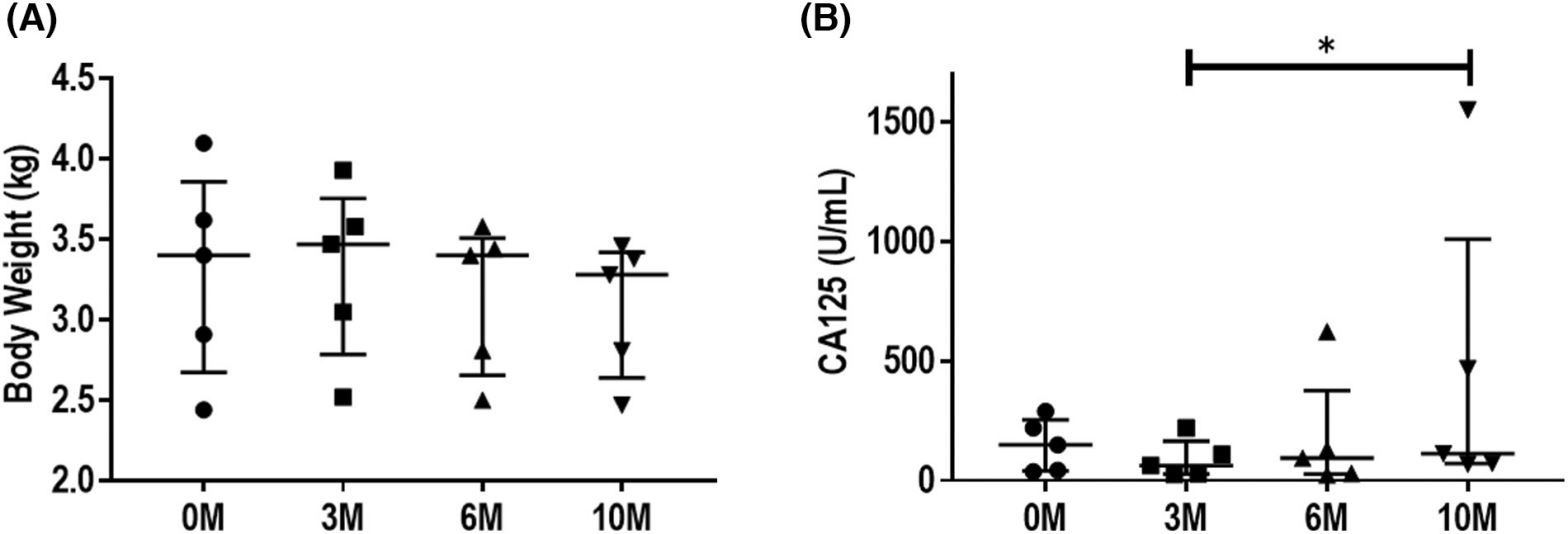
Comparison of body weight (A) and CA125 levels (B) of the monkeys via one‐way analysis of variance, followed by the Dunn test. *p < 0.05. CA125, cancer antigen 125.

Table 2 shows the body weight changes of the monkeys during the experiment. The median body weight in the intervention group was 3.4 kg before the intervention, 3.5 kg at 3 months, 3.4 kg at 6 months, and 3.3 kg at 10 months (Figure 5A). The median body weight in the control group was 3.7 kg before the experiment, 3.6 kg at 3 months, 3.5 kg at 6 months, and 3.5 kg at 10 months. No significant changes were observed in either group during the study.

**TABLE 2:** Body weight change in monkeys in this study.

	Identification number	0 M	3 M	6 M	10 M
Intervention group	I‐1	4.1	3.9	3.4	3.5
	I‐2	2.4	2.5	2.5	2.5
	I‐3	2.9	3.1	2.8	2.8
	I‐4	3.4	3.6	3.6	3.3
	I‐5	3.6	3.5	3.4	3.4
Control group	C‐1	4.1	4.1	4.0	3.9
	C‐2	3.2	3.0	3.0	3.0

Abbreviation: M, months.

Table 3 shows the changes in CA125 levels in monkeys during this experiment. The median CA125 level in the intervention group was 150 U/mL before the intervention, 63 U/mL at 3 months, 94 U/mL at 6 months, and 113 U/mL at 10 months. The median CA125 level in the control group was 16 U/mL before the experiment, 19 U/mL at 3 months, 12 U/mL at 6 months, and 16 U/mL at 10 months. Although no change occurred over time in the control group, CA125 levels in the intervention group differed significantly between three and 10 months (*p* = 0.02; Figure 5B).

**TABLE 3:** Change in CA125 levels (U/mL) in monkeys in this study.

	Identification number	0 M	3 M	6 M	10 M
Intervention group	I‐1	289.0	220.0	622.0	1550.0
	I‐2	220.0	110.0	130.0	468.0
	I‐3	42.9	24.8	23.0	113.0
	I‐4	150.0	63.1	94.0	73.0
	I‐5	37.8	29.1	31.0	68.9
Control group	C‐1	10.2	17.9	10.0	11.6
	C‐2	22.1	20.9	14.0	21.2

Abbreviation: M, months.

## 4 DISCUSSION

To our knowledge, no previous study has reported MSC administration in monkeys with endometriosis. Weekly intravenous UC‐MSC administration for 3 months decreased CA125 levels in all monkeys; however, laparoscopic findings improved in only one monkey. Subsequent weekly intravenous administration combined with once‐monthly intraperitoneal administration for 3 months was unsuccessful in alleviating endometriosis. Finally, after a 1‐month treatment‐free interval, 3 months of weekly intraperitoneal injections without intravenous administration exacerbated endometriosis.

Endometriosis induces the release of pro‐inflammatory and anti‐inflammatory cytokines in the pelvic cavity.^21^, ^22^ Pro‐inflammatory cytokines, such as interleukin (IL)‐1, IL‐6, and tumor necrosis factor‐alpha, have been detected in patients with endometriosis.^23^, ^24^ These inflammatory cytokines are related to infertility and pain^23^, ^25^ and promote the presence and extent of endometriosis.^23^ Among anti‐inflammatory cytokines, IL‐4, IL‐10, and transforming growth factor‐beta (TGF‐β) levels are also elevated both in the peripheral blood and in the peritoneal fluid of patients with endometriosis.^26^, ^27^, ^28^, ^29^ These anti‐inflammatory cytokines contribute to the development of endometriosis via immune escape, cell proliferation, adhesion, migration, invasion, epithelial–mesenchymal transition, and angiogenesis.^22^

Opposing inflammatory effectors are observed during endometriosis. Therefore, using immunomodulatory agents as therapeutic candidates for endometriosis has remained challenging. Among MSCs with demonstrated immunomodulatory effects,^30^ UC‐MSCs offer several advantages: they can be isolated directly from the UC without requiring invasive procedures^31^ and show good clinical efficacy and biocompatibility.^32^, ^33^, ^34^, ^35^, ^36^ Therefore, in light of their anti‐proliferative effects on endometrial cells in vitro,^5^, ^6^ we expected UC‐MSCs to serve as potential effective agents against endometriosis in vivo. Intravenous US‐MSC administration reduced the CA125 levels in all monkeys; however, laparoscopy showed improvement in endometrial lesions in only one monkey. Therefore, we performed intraperitoneal administration to observe a direct effect. However, both laparoscopic findings and CA125 levels worsened after intraperitoneal administration. In particular, for CA125, a statistically significant difference was observed despite the small number of samples used in our study. These results were consistent with the findings of Abomary et al., who used endometrium‐derived MSCs.^9^

Anti‐inflammatory cytokines, such as TGF‐β, are involved in extending endometriotic lesions. Notably, MSCs are also known to secrete TGF‐β.^37^, ^38^, ^39^ Specifically, UC‐MSCs produce more TGF‐β than do MSCs derived from amniotic membrane and adipose tissue.^38^ The level of activated TGF‐β, which induces regulatory T cells effectively, is elevated in ascites in patients with endometriosis.^29^ MSCs derived from endometriotic lesions significantly promote fibrogenesis via TGF‐β and WNT1.^11^ Furthermore, TGF‐β induces fibrosis during endometriosis.^40^ Therefore, anti‐inflammatory cytokines, including TGF‐β secreted by UC‐MSCs, likely contribute to the exacerbation of endometriosis by intraperitoneal administration.

A limitation of this study was the small sample size. However, obtaining enough monkeys with spontaneous endometriosis for statistical analysis is not easy. Therefore, the decrease in CA125 levels after intravenous UC‐MSC administration may have reached statistical significance if the sample size was larger. In addition, only two monkeys were included in the control group, which was insufficient to demonstrate the natural change in the ASRM score and CA125 levels in monkeys with endometriosis. However, the exacerbation of laparoscopic findings noted in this study was not observed in our previous report.^13^ In addition, the high CA125 levels noted in this study were not demonstrated previously.^19^ Therefore, we considered that the changes in the ASRM score and CA125 levels were not within the normal range. Second, unlike rodents, the genetic backgrounds of the monkeys were different because they were not inbred. Third, this was a xenograft experiment; therefore, the administered human cells are highly likely to be immunologically rejected by the monkey host, thereby provoking inflammatory responses that could worsen endometriosis. However, in previous experiments wherein human MSCs were administered to monkeys, no xenograft‐induced inflammation was observed, regardless of the administration method (Table 4).^41^, ^42^, ^43^, ^44^, ^45^, ^46^, ^47^ Wang et al.,^42^ Feng et al.,^12^ and Yeung et al.,^47^ administered human MSCs to healthy monkeys and proved the safety of xenografts involving human MSCs. In particular, Feng et al. showed the efficacy of local administration of these cells into the brain, instead of intravascular administration. Therefore, MSCs were used undiluted, without adverse effects. Furthermore, Guo et al.^45^ reported that intraperitoneal human UC‐MSC administration was effective for acute liver failure. In addition to monkeys, Perry et al.^48^ locally administered human UC‐MSCs to rodents, and Chu et al.^49^ intratracheally injected UC‐MSCs into a rat model of pulmonary fibrosis. Both experiments revealed their efficacy without side effects. As UC‐MSCs do not express HLA‐DR and have an immune escape mechanism, they do not cause inflammation, even in a murine model. Taken together, we do not consider that the exacerbation of endometriosis was caused by xenograft‐induced inflammation after intraperitoneal administration of human UC‐MSCs. We consider it reasonable to assume that the paracrine effect produced by UC‐MSCs exacerbated endometriosis. Fourth, there are no data available on the number of cells remaining in the blood vessels or peritoneal cavity and the duration of their presence. However, even if MSCs disappeared in a few days, cytokines such as TGF‐β secreted by MSCs in the peritoneal cavity might exacerbate endometriosis. As the present study protocol was based on weekly administration, we considered that endometriosis was constantly induced by these cytokines.

**TABLE 4:** Xenograft experiment using human MSC in monkeys.

First author	Year	Administered cells	Method of administration to monkeys	Target disease	Result	Side effect
Li J	2010	Human BM‐MSCs	Local injection in the brain	Cerebral ischemic injury	Effective	None
Wang Y	2012	Human UC‐MSCs	I.V.	To evaluate the safety of hMSC transfusion with repeated administration	Safety	No adverse effect
Feng M	2014	Human BM‐MSCs	Intracerebral transplantation	To evaluate the safety of hMSCs transplanted in the brain of healthy monkeys	Safety	No adverse effect
Fernandez‐Pernas P	2017	Human SM‐MSCs	I.V.	Osteoarthritis	Effective	None
Lee KW	2018	Human BM‐MSCs	Injection to the suprarenal aorta	Renal ischemia–reperfusion injury	Effective	None
Guo G	2019	Human UC‐MSCs	I.P.	Acute liver failure	Effective	None
Liu S	2019	Human UC‐MSCs	I.V.	Multiple sclerosis	Effective	None
Yeung CK	2022	Human UC‐MSC spheroids	I.V.	To evaluate the safety of hMSC spheroids transplanted into healthy monkeys	Safety	No adverse effect

Abbreviations: BM‐MSCs, bone marrow‐derived mesenchymal stem cells; I.P., intraperitoneal injection; I.V., intravenous injection; MSCs, mesenchymal stem cells; SM‐MSCs, synovial membrane‐derived MSCs; UC‐MSCs, umbilical cord‐derived mesenchymal stem cells.

In conclusion, intraperitoneal UC‐MSC administration significantly exacerbated endometriosis in cynomolgus monkeys. Although the potential efficacy of intravenous UC‐MSC administration in endometriosis remains unclear, our findings in a nonhuman primate model of endometriosis suggested that intraperitoneal UC‐MSC administration should not be used to treat endometriosis.

## CONFLICT OF INTEREST STATEMENT

Author T. Murakami is an Editorial Board member of *Reproductive Medicine and Biology* and a co‐author of this article. To remove bias, he was excluded from all editorial decision‐making related to the acceptance of this article for publication.

## ETHICS STATEMENT

The animal study was conducted at the Research Center for Animal Life Science at Shiga University of Medical Science with permission from the Animal Care and Use Committee of Shiga University of Medical Science (approval number: 2021‐3‐8). Umbilical cords (UCs) were obtained in a manner approved by the Ethics Committees of the Institute of Medical Science, University of Tokyo, NTT Medical Center Hospital, and Yamaguchi Hospital, Japan (approval number: 27‐61‐1223, 31‐2). UCs were collected after informed consent was obtained from pregnant women planning to undergo a cesarean section.

## HUMAN RIGHTS STATEMENT AND IMPROVED CONSENT

All procedures involving humans were in accordance with the ethical standards of the responsible committee on human experimentation (institutional and national) and with the Helsinki Declaration of 1964 and its later amendments. Informed consent was obtained from all patients included in the study.

## ANIMAL STUDIES

All institutional and national guidelines for the care and use of laboratory animals were followed. This study was conducted in strict accordance with the Fundamental Guidelines for Proper Conduct of Animal Experiments and Related Activities in Academic Research Institutions, regulated by the Ministry of Education, Culture, Sports, Science, and Technology, Japan.

## Supporting information


Figure S1.

## Data Availability

The data that support the findings of this study are available upon reasonable request from the corresponding author.
