# Supplementary figures and images for: Impact of administering umbilical cord‐derived mesenchymal stem cells to cynomolgus monkeys with endometriosis
Source: Reprod Med Biol. 2023 Sep 8;22(1):e12540. doi: 10.1002/rmb2.12540 (PMC10491929; doi:10.1002/rmb2.12540)

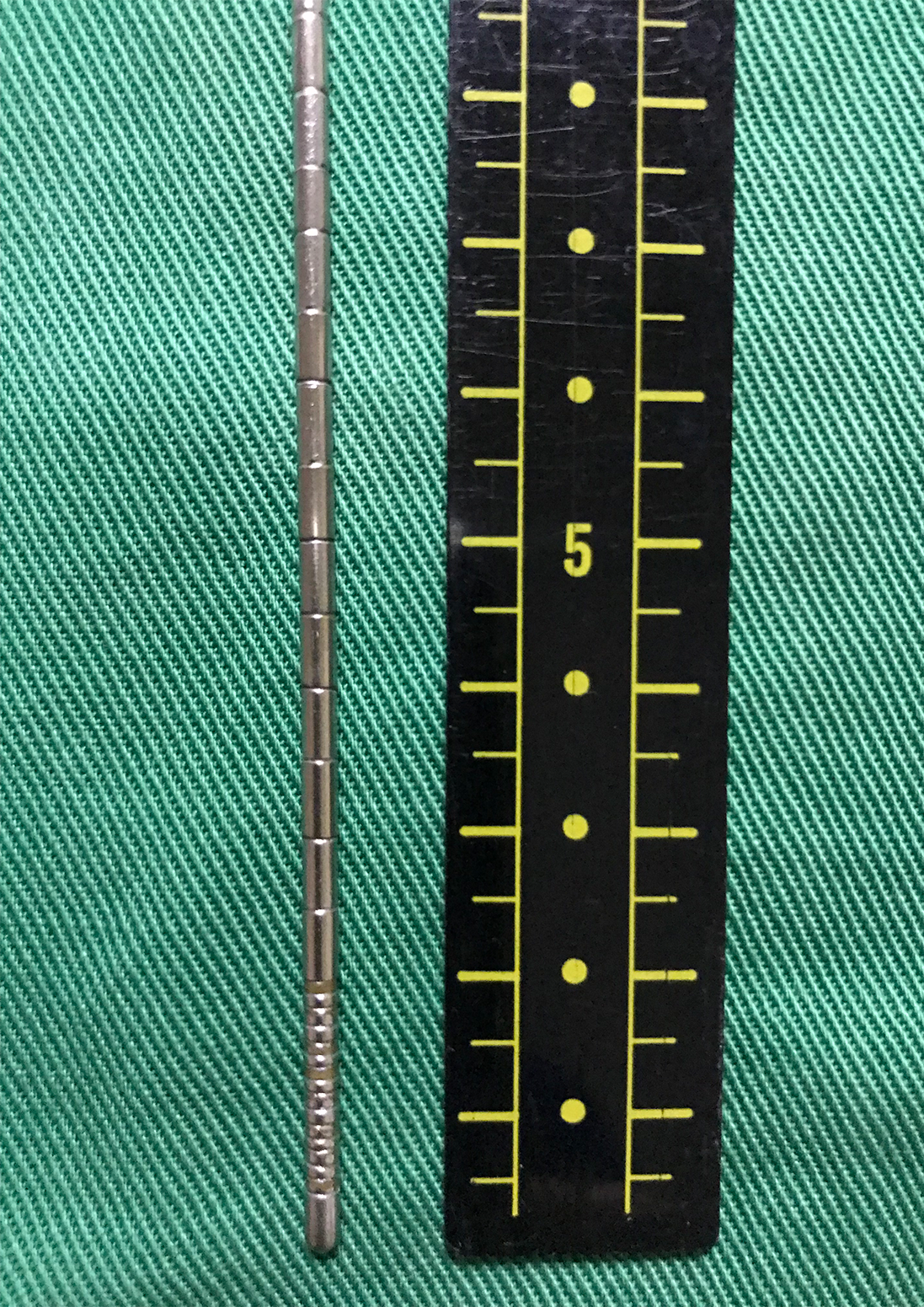

Supplement: Supplementary file 1 — Figure S1. [file RMB2-22-e12540-s001.tif]
